# 1,1′-Methyl­enebis(4,4′-bipyridin-1-ium) dibromide

**DOI:** 10.1107/S2414314622005260

**Published:** 2022-05-20

**Authors:** Sara A. Schuster, Volodymyr V. Nesterov, Bradley W. Smucker

**Affiliations:** a Austin College, 900 N Grand, Sherman, TX 75090, USA; b Department of Chemistry, University of North Texas, 1508 W. Mulberry, Denton, TX, 76201, USA; University of Antofagasta, Chile

**Keywords:** crystal structure, pyridinium, hydrogen bonding, π–π inter­actions

## Abstract

The asymmetric unit of the title salt, C_21_H_18_N_4_^2+^·2Br^−^, comprises half of the mol­ecule and a bromide ion. The chevron-shaped cations stack as columns in the [001] direction with suitable inter­molecular distance for π–π inter­actions. These cationic columns are further stabilized by inter­columnar C—H⋯N hydrogen bonding with the bromide ions distributed between them.

## Abstract 2

The asymmetric unit of the title salt comprises half of the mol­ecule and a bromide ion. The chevron-shaped cations stack as columns that are stabilized by through space electrostatic inter­actions and inter­columnar hydrogen bonding.

## Graphical abstract

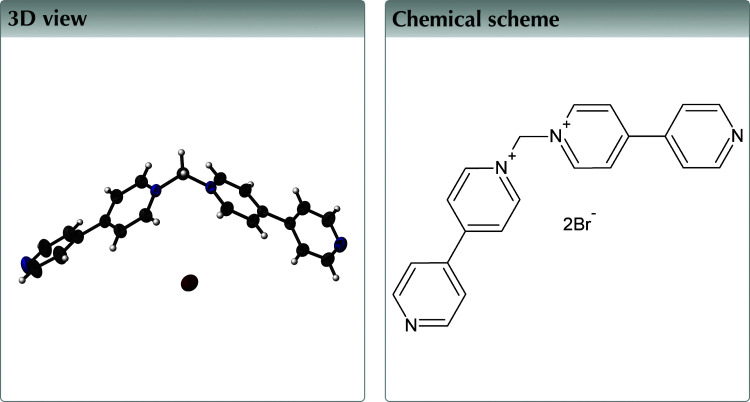

## Structure description

**Figure 1:**
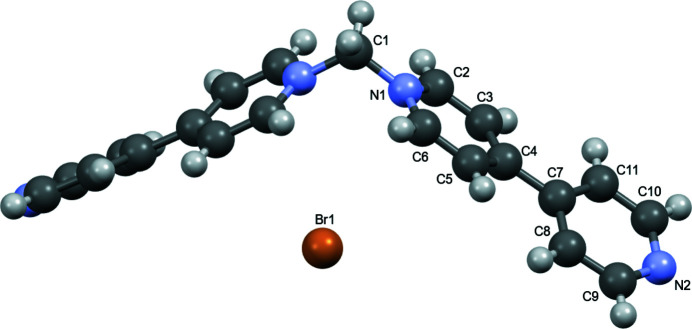
Ellipsoid (50%) representation of the title complex with the cation expanded by symmetry.

**Figure 2:**
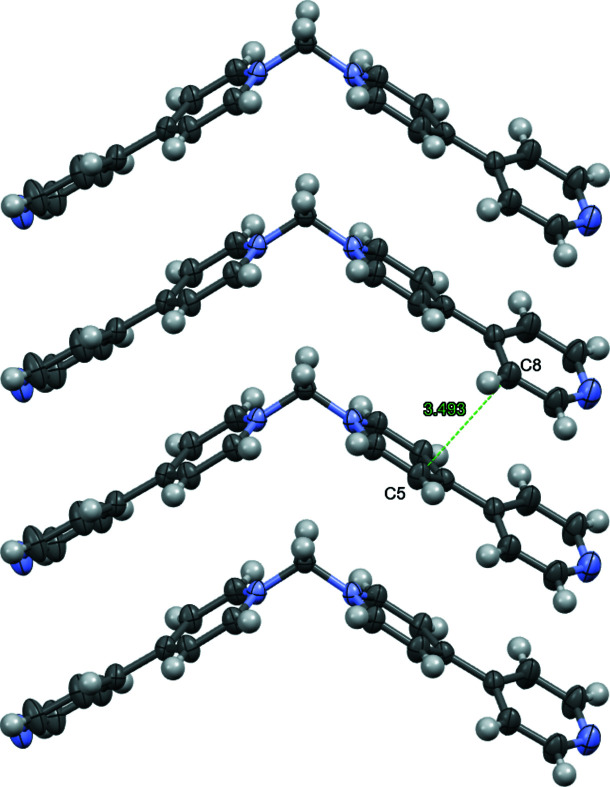
Ellipsoid (50%) representation of the columnar stacking of the cations with distance between C5 and C8(*x*, *y*, *z* + 1) shown. Bromide ions are omitted for clarity.

**Figure 3:**
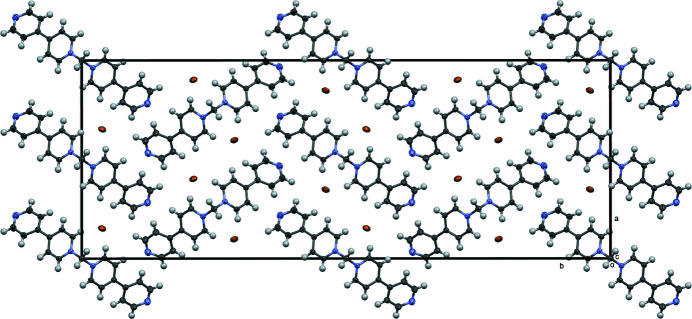
View down the crystallographic *c* axis showing the distribution of bromide ions (brown) between the columns of cations. Cell axes shown with ellipsoid (50%) representation.

**Figure 4:**
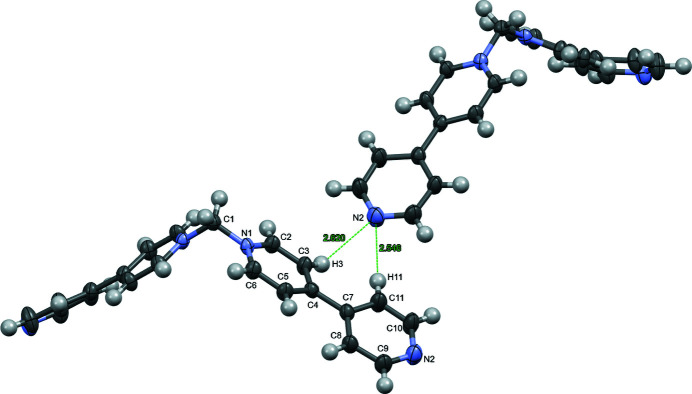
Ellipsoid (50%) representation of the inter-columnar N⋯H distances between H3 and H11 atoms of the bipyridinium and the N2(− 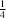 + *x*, 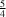 − *y*, 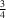 + *z*) atom on the terminal pyridyl ring. Bromide ions are omitted for clarity.

The N1—C1—N1(1 − *x*, 1 − *y*, *z*) bond angle of the chevron-shaped 1,1′-methyl­enebis-4,4′-bipyridinium cation in the title compound (Fig. 1 ▸) is 111.1 (4)°, which is slightly smaller than the angle of 112.3 (4)° in the corresponding PF_6_^−^ salt (Blanco *et al.*, 2007 ▸). The packing resulting from the smaller bromide results in the cations of the title compound stacking to form columns (Fig. 2 ▸) in the [001] direction with the bromide ions distributed between them (Fig. 3 ▸). The closest inter­molecular C⋯C distance between these stacked cations is 3.493 (5) Å between C5 and C8(*x*, *y*, 1 + *z*), which is indicative of through space electrostatic inter­actions (Martinez & Iverson, 2012 ▸). The structure of the aforementioned PF_6_^−^ salt does not form these stacked columns. Even with bromide ions, the structure of the slightly larger 1,1′-methyl­enebis{4-[(*E*)-2-(pyridin-4-yl)vin­yl]pyridinium} dibromide dihydrate packs in back-to-back zigzag ribbons (Neal *et al.*, 2022 ▸) instead of the columns seen in this structure. For the title compound, in the extended structure, the columns of the cation are positioned such that the H3 and H11 atoms of the bipyridinium moiety are 2.620 and 2.546 Å, respectively, from the N2(− 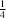 + *x*, 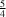 − *y*, 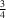 + *z*) atom of a pyridyl group in an adjacent column (Fig. 4 ▸). The shorter N⋯H distance for H11 results from the rotation of the pyridyl ring relative to the pyridinium ring by 21.00 (14)° [dihedral angle between the planes of the pyridinium (N1/C2–C6) and pyridyl (N2/C7–C11) rings].

## Synthesis and crystallization

The title compound was synthesized following published procedures (Blanco *et al.*, 2007 ▸). Colorless block-shaped crystals were grown from the vapor diffusion of THF into a DMF solution of the compound.

## Refinement

Crystal data, data collection and structure refinement details are summarized in Table 1 ▸.

## Supplementary Material

Crystal structure: contains datablock(s) I. DOI: 10.1107/S2414314622005260/bx4020sup1.cif

Structure factors: contains datablock(s) I. DOI: 10.1107/S2414314622005260/bx4020Isup2.hkl

Supporting information file. DOI: 10.1107/S2414314622005260/bx4020Isup3.mol

Supporting information file. DOI: 10.1107/S2414314622005260/bx4020Isup4.cml

CCDC reference: 2173318

Additional supporting information:  crystallographic information; 3D view; checkCIF report

## Figures and Tables

**Table 1:** Experimental details

Crystal data	
Chemical formula	C_21_H_18_N_4_^2+^·2Br^−^
*M*_r_	486.21
Crystal system, space group	Orthorhombic, *F* *d* *d*2
Temperature (K)	220
*a*, *b*, *c* (Å)	18.0776 (2), 48.2301 (5), 4.5424 (2)
*V* (Å^3^)	3960.45 (18)
*Z*	8
Radiation type	Cu *K*α
μ (mm^−1^)	5.29
Crystal size (mm)	0.04 × 0.02 × 0.01

Data collection	
Diffractometer	XtaLAB Synergy, Dualflex, HyPix
Absorption correction	Multi-scan *CrysAlis PRO* (Rigaku OD, 2021 ▸)
*T*_min_, *T*_max_	0.775, 1.000
No. of measured, independent and observed [*I* > 2σ(*I*)] reflections	21913, 2127, 2118
*R*_int_	0.028
(sin θ/λ)_max_ (Å^−1^)	0.639

Refinement	
*R*[*F* ^2^ > 2σ(*F* ^2^)], *wR*(*F* ^2^), *S*	0.022, 0.053, 1.16
No. of reflections	2127
No. of parameters	123
No. of restraints	1
H-atom treatment	H-atom parameters constrained
Δρ_max_, Δρ_min_ (e Å^−3^)	0.26, −0.26
Absolute structure	Flack *x* determined using 895 quotients [(*I* ^+^)−(*I* ^−^)]/[(*I* ^+^)+(*I* ^−^)] (Parsons *et al.*, 2013 ▸)
Absolute structure parameter	−0.015 (7)

Computer programs: *CrysAlis PRO* (Rigaku OD, 2021 ▸), *SHELXT2018/2* (Sheldrick, 2015*a* ▸), *SHELXL2018/3* (Sheldrick, 2015*b* ▸), *Mercury* (Macrae *et al.*, 2020 ▸), and *OLEX2* (Dolomanov *et al.*, 2009 ▸).

## full crystallographic data

### Crystal data

**Table:**

C_21_H_18_N_4_^2+^·2Br^−^	*D*_x_ = 1.631 Mg m^−^^3^
*M**_r_* = 486.21	Cu *K*α radiation, λ = 1.54178 Å
Orthorhombic, *F**d**d*2	Cell parameters from 18629 reflections
*a* = 18.0776 (2) Å	θ = 3.7–78.7°
*b* = 48.2301 (5) Å	µ = 5.29 mm^−^^1^
*c* = 4.5424 (2) Å	*T* = 220 K
*V* = 3960.45 (18) Å^3^	Block, clear light colourless
*Z* = 8	0.04 × 0.02 × 0.01 mm
*F*(000) = 1936	

### Data collection

**Table:**

XtaLAB Synergy, Dualflex, HyPix diffractometer	2127 independent reflections
Radiation source: micro-focus sealed X-ray tube, PhotonJet (Cu) X-ray Source	2118 reflections with *I* > 2σ(*I*)
Mirror monochromator	*R*_int_ = 0.028
Detector resolution: 10.0000 pixels mm^-1^	θ_max_ = 79.9°, θ_min_ = 3.7°
ω scans	*h* = −22→22
Absorption correction: multi-scan CrysAlisPro (Rigaku OD, 2021)	*k* = −60→59
*T*_min_ = 0.775, *T*_max_ = 1.000	*l* = −5→5
21913 measured reflections	

### Refinement

**Table:**

Refinement on *F*^2^	Hydrogen site location: inferred from neighbouring sites
Least-squares matrix: full	H-atom parameters constrained
*R*[*F*^2^ > 2σ(*F*^2^)] = 0.022	*w* = 1/[σ^2^(*F*_o_^2^) + (0.0076*P*)^2^ + 10.8403*P*] where *P* = (*F*_o_^2^ + 2*F*_c_^2^)/3
*wR*(*F*^2^) = 0.053	(Δ/σ)_max_ = 0.002
*S* = 1.16	Δρ_max_ = 0.26 e Å^−^^3^
2127 reflections	Δρ_min_ = −0.25 e Å^−^^3^
123 parameters	Absolute structure: Flack *x* determined using 895 quotients [(*I*^+^)-(*I*^-^)]/[(*I*^+^)+(*I*^-^)] (Parsons *et al.*, 2013)
1 restraint	Absolute structure parameter: −0.015 (7)

### Special details

**Table:**

Geometry. All esds (except the esd in the dihedral angle between two l.s. planes) are estimated using the full covariance matrix. The cell esds are taken into account individually in the estimation of esds in distances, angles and torsion angles; correlations between esds in cell parameters are only used when they are defined by crystal symmetry. An approximate (isotropic) treatment of cell esds is used for estimating esds involving l.s. planes.

### Fractional atomic coordinates and isotropic or equivalent isotropic displacement parameters (Å^2^)

**Table:**

	*x*	*y*	*z*	*U*_iso_*/*U*_eq_	Occ. (<1)
Br1	0.65219 (2)	0.46137 (2)	0.25829 (7)	0.04764 (12)	
N1	0.53731 (13)	0.52084 (5)	0.8226 (6)	0.0304 (6)	
N2	0.71760 (17)	0.62446 (6)	−0.0002 (10)	0.0532 (8)	
C1	0.500000	0.500000	1.0054 (12)	0.0327 (8)	
H1A	0.463945	0.509067	1.130882	0.039*	0.5
H1B	0.536051	0.490934	1.130908	0.039*	0.5
C2	0.49878 (16)	0.54322 (6)	0.7299 (10)	0.0360 (7)	
H2	0.449003	0.545007	0.778057	0.043*	
C3	0.53289 (17)	0.56327 (6)	0.5653 (8)	0.0370 (8)	
H3	0.506094	0.578632	0.502375	0.044*	
C4	0.60776 (15)	0.56086 (5)	0.4907 (10)	0.0318 (6)	
C5	0.64502 (17)	0.53725 (7)	0.5872 (8)	0.0383 (8)	
H5	0.694617	0.534836	0.539168	0.046*	
C6	0.60952 (15)	0.51762 (6)	0.7513 (10)	0.0365 (6)	
H6	0.635047	0.501967	0.814337	0.044*	
C7	0.64574 (17)	0.58273 (6)	0.3206 (8)	0.0353 (8)	
C8	0.71058 (19)	0.57758 (7)	0.1703 (9)	0.0425 (8)	
H8	0.731788	0.560015	0.174291	0.051*	
C9	0.7435 (2)	0.59865 (7)	0.0146 (12)	0.0515 (9)	
H9	0.786861	0.594616	−0.087262	0.062*	
C10	0.6558 (2)	0.62938 (8)	0.1498 (11)	0.0561 (12)	
H10	0.637202	0.647358	0.148926	0.067*	
C11	0.6175 (2)	0.60960 (7)	0.3062 (11)	0.0492 (10)	
H11	0.573509	0.614083	0.400858	0.059*	

### Atomic displacement parameters (Å^2^)

**Table:**

	*U*^11^	*U*^22^	*U*^33^	*U*^12^	*U*^13^	*U*^23^
Br1	0.03445 (16)	0.0558 (2)	0.0527 (2)	0.01006 (15)	−0.00518 (18)	0.0093 (2)
N1	0.0247 (11)	0.0267 (11)	0.0400 (16)	−0.0010 (9)	−0.0013 (10)	−0.0019 (10)
N2	0.0482 (16)	0.0388 (14)	0.073 (2)	−0.0034 (12)	0.015 (2)	0.0040 (19)
C1	0.0306 (18)	0.0320 (18)	0.035 (2)	−0.0004 (15)	0.000	0.000
C2	0.0255 (13)	0.0324 (14)	0.0500 (19)	0.0050 (11)	0.0050 (16)	0.0038 (15)
C3	0.0287 (15)	0.0304 (14)	0.052 (2)	0.0047 (11)	0.0043 (14)	0.0056 (14)
C4	0.0273 (13)	0.0283 (12)	0.0396 (16)	−0.0010 (10)	0.0011 (15)	−0.0045 (16)
C5	0.0233 (14)	0.0355 (16)	0.056 (2)	0.0030 (11)	0.0061 (13)	0.0021 (14)
C6	0.0245 (12)	0.0331 (14)	0.0520 (18)	0.0053 (10)	0.0012 (16)	0.0047 (16)
C7	0.0315 (15)	0.0295 (13)	0.045 (2)	−0.0018 (11)	0.0014 (13)	−0.0021 (13)
C8	0.0353 (17)	0.0331 (16)	0.059 (2)	0.0028 (13)	0.0115 (15)	−0.0021 (14)
C9	0.0421 (18)	0.0399 (16)	0.073 (3)	−0.0003 (13)	0.021 (2)	−0.001 (2)
C10	0.050 (2)	0.0330 (17)	0.085 (3)	0.0041 (15)	0.022 (2)	0.0093 (18)
C11	0.0384 (17)	0.0357 (16)	0.073 (3)	0.0041 (13)	0.0183 (19)	0.0022 (18)

### Geometric parameters (Å, º)

**Table:**

N1—C1	1.468 (4)	C4—C7	1.477 (4)
N1—C2	1.352 (4)	C5—H5	0.9300
N1—C6	1.354 (4)	C5—C6	1.365 (5)
N2—C9	1.332 (4)	C6—H6	0.9300
N2—C10	1.330 (5)	C7—C8	1.379 (4)
C1—H1A	0.9700	C7—C11	1.394 (4)
C1—H1B	0.9700	C8—H8	0.9300
C2—H2	0.9300	C8—C9	1.374 (5)
C2—C3	1.369 (5)	C9—H9	0.9300
C3—H3	0.9300	C10—H10	0.9300
C3—C4	1.400 (4)	C10—C11	1.376 (5)
C4—C5	1.394 (4)	C11—H11	0.9300
			
C2—N1—C1	119.1 (2)	C6—C5—C4	120.7 (3)
C2—N1—C6	120.9 (3)	C6—C5—H5	119.6
C6—N1—C1	120.0 (2)	N1—C6—C5	120.3 (3)
C10—N2—C9	115.9 (3)	N1—C6—H6	119.9
N1—C1—N1^i^	111.1 (4)	C5—C6—H6	119.9
N1—C1—H1A	109.4	C8—C7—C4	121.7 (3)
N1^i^—C1—H1A	109.4	C8—C7—C11	117.1 (3)
N1—C1—H1B	109.4	C11—C7—C4	121.2 (3)
N1^i^—C1—H1B	109.4	C7—C8—H8	120.3
H1A—C1—H1B	108.0	C9—C8—C7	119.4 (3)
N1—C2—H2	119.9	C9—C8—H8	120.3
N1—C2—C3	120.1 (3)	N2—C9—C8	124.4 (3)
C3—C2—H2	119.9	N2—C9—H9	117.8
C2—C3—H3	119.7	C8—C9—H9	117.8
C2—C3—C4	120.6 (3)	N2—C10—H10	117.9
C4—C3—H3	119.7	N2—C10—C11	124.3 (3)
C3—C4—C7	121.1 (3)	C11—C10—H10	117.9
C5—C4—C3	117.3 (3)	C7—C11—H11	120.5
C5—C4—C7	121.6 (3)	C10—C11—C7	119.0 (3)
C4—C5—H5	119.6	C10—C11—H11	120.5
			
N1—C2—C3—C4	0.1 (6)	C4—C7—C8—C9	179.8 (4)
N2—C10—C11—C7	2.5 (8)	C4—C7—C11—C10	178.7 (4)
C1—N1—C2—C3	178.4 (4)	C5—C4—C7—C8	20.9 (6)
C1—N1—C6—C5	−178.5 (4)	C5—C4—C7—C11	−158.9 (4)
C2—N1—C1—N1^i^	87.1 (3)	C6—N1—C1—N1^i^	−93.5 (3)
C2—N1—C6—C5	1.0 (6)	C6—N1—C2—C3	−1.0 (6)
C2—C3—C4—C5	0.9 (6)	C7—C4—C5—C6	178.1 (4)
C2—C3—C4—C7	−178.2 (4)	C7—C8—C9—N2	0.9 (7)
C3—C4—C5—C6	−0.9 (6)	C8—C7—C11—C10	−1.1 (6)
C3—C4—C7—C8	−160.1 (4)	C9—N2—C10—C11	−2.1 (8)
C3—C4—C7—C11	20.1 (6)	C10—N2—C9—C8	0.3 (7)
C4—C5—C6—N1	0.1 (6)	C11—C7—C8—C9	−0.4 (6)

Symmetry code: (i) −*x*+1, −*y*+1, *z*.
